# Learning objectives: an epiphany

**DOI:** 10.1002/2211-5463.13328

**Published:** 2021-12-01

**Authors:** Priscilla Soulié, Pierre Cosson

**Affiliations:** ^1^ Faculty of Medicine University of Geneva Switzerland

## Abstract

A new Bachelor–Master curriculum in Biomedical Sciences was created at the University of Geneva in 2017. As we organized the new curriculum, we discovered the usefulness of learning objectives. This goal‐oriented approach of teaching proved essential to determine the overall structure of the teaching program, as well as the content of specific courses, and the nature of the examinations. It led us to include innovative elements in the program, preparing students for real‐life situations. Finally, it convinced us to change our role as teachers, in order to engage students in a more active learning relationship.

## Graphical abstract

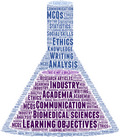

Back in 2010, we started organizing a new Bachelor–Master curriculum at the University of Geneva. Over a period of five years, this program prepares life science students to join either an academic research laboratory or a private company. The project evolved, driven by a number of motivated colleagues, over periods of ups and downs, until September 2017 when the first class of students joined the newly created Bachelor in Biomedical Sciences. Since then, the project has progressed well, with student enrollment increasing steadily. The first class entered the fifth and final year of the program (2nd year of Master) in September 2021.

In retrospect, if we had to name one single thing that the creation of this curriculum taught us, it would be that learning objectives are fantastic tools. What we want to share with you in this text is our epiphany with respect to the meaning and the power of learning objectives. This realization helped structure the whole five‐year program. It was key to defining the required knowledge basis, the professional skills, the learning methods, and even the human skills that should be developed by students. In the end, it profoundly transformed our understanding of our own role as teachers.

## A staggering amount of knowledge

Somewhere in an imaginary library, there is a huge encyclopedia containing all the accumulated knowledge in the field of life sciences. Open this encyclopedia at any page, stop the first ten people who enter the library, including eminent professors, and question them on the content of that page. They will most probably not be able to offer useful answers. How could they? The amount of knowledge in life sciences is staggering, and growing at an ever‐increasing rate. You could scale down your expectations and quiz your victims only on established knowledge found in reference textbooks, but the result would probably be the same. No one has complete knowledge of every area of biomedical sciences, and no one person can completely cover the more restricted field of biological sciences, or even the subfield of cellular biology. So how much should life science students learn, and how much should they retain? Why make students learn things that they will mostly forget later? Is it a way of testing their motivation and learning capacities, based on mostly useless knowledge? Will this half‐forgotten knowledge form the indispensable basis for scientific culture, a half‐intuitive understanding of the major issues in life sciences? And if so, who would decide what these major issues would be?

One can choose to ignore the most arcane of these questions, but when creating a new curriculum, one is repeatedly confronted with the most trivial of them: How many hours of study should be devoted to what subject? Is a general understanding of each topic sufficient or should it be studied in‐depth? Is theoretical knowledge appropriate, or should it be backed with practical elements (e.g., laboratory work)? In the end, what are we trying to teach? What are the learning objectives for the students?

## The power of learning objectives

Our first encounter with learning objectives was not a happy one. As teachers, we had taken courses in pedagogy, and on several occasions, we were asked by pedagogues to define the objectives of our teaching. It made us feel a bit silly. To the question ‘what is the learning objective of your course?’, we would answer ‘that the students know the structure of the endoplasmic reticulum, or renal physiology etc.’. The pedagogues insisted: ‘what do you expect them to be able to do with that knowledge? You must name an action, something that they can do’. Our best answer was still pathetic: ‘the students must be able to answer questions relative to the structure of the endoplasmic reticulum’. We were going nowhere and felt that we were missing the point entirely.

When ten years later, we found ourselves confronted with the question of what to include or exclude in the emerging biomedical sciences curriculum, the question of learning objectives surfaced again, but this time we saw it as essential and meaningful. In truth, learning objectives mostly make sense if you consider the finality of a big chunk of teaching. Defining learning objectives for a whole Bachelor–Master program was ideal. This is the story of how it went for us.

At the end of their studies, what should the young graduates be able to do? In our case, they should be able to work in a life science company or in an academic research laboratory. An analysis of the local job market over 4 months (429 job offers) indicated that the vast majority of positions open to life scientists were in companies (94%), for professionals with at least a Master level (75%), and with a strong focus on development of health products (51%), biotechnological production (27%), or applied research (10%). Acknowledging this, to complement our understanding of the academic world, we interviewed a large number of professionals working in companies recruiting life scientists. Their answers stressed five main points: 1—Students should have a strong scientific background, 2—with a focus on biomedical knowledge. 3—They should be able to communicate efficiently (in English). 4—They should have a global understanding of the development of health products. 5—They should be familiar with the world of companies and academic research laboratories.

From this list, we derived a series of broad learning objectives. Here is one inspired by points 1, 2 (scientific and biomedical knowledge), and 3 (communication): Young graduates should be able to read a biomedical scientific document, write a summary, make an oral presentation, provide a critical assessment of the results and conclusions, and define new perspectives. These skills were then broken down into more focused learning objectives that were included in the different years of the program. For example, to ensure that students can perform high‐level scientific analysis and communication at the end of the Master, in addition to providing fundamental biomedical knowledge the program should include a series of courses focused on the basic required abilities: statistics, English language, oral skills, and writing skills. When designing each of these courses, it was very helpful to keep in mind the final aim, to separate useful from useless knowledge (for these students). Statistics, for example, are essential tools, but should be included only inasmuch as they are necessary to evaluate experimental data.

In addition to acquisition of the basic skills and knowledge, the program should provide for practice of the required skills: reading and analyzing scientific articles. For these activities, a gradual acquisition of skills should be planned: The first articles that are studied are simpler than the last ones. A typical introductory Bachelor course dissects in great detail a relatively simple article, whereas a Master course proposes to compare within one single session two complex articles with divergent conclusions.

A similar process led us to define a few other general objectives: The students should be able to propose and execute a research program, or to describe and analyze a drug/medical device development program. This led us to insert a wide collection of courses in the program, including those focusing on practical laboratory work, ethics, law, drug discovery and development, and medical devices.

Defining learning objectives precisely allowed us to structure the whole program around them in a coherent way: the overall structure of the learning program, a list of necessary courses, how far to go and what learning methods to use within each course. It also defined how the examinations should be organized. It is so obvious: If you want students to be able to do something, teach them the required basics, then make them do it! To evaluate the students, just make them do it one more time and assess how well they manage. That is the power of learning objectives. It took us a decade to figure it out.

## ‘This is not a drill’

An adequate knowledge basis and skill set is probably not sufficient to transform a student into a player in the professional world. When we were five years old, we all sat on a little chair in front of the first of a long series of schoolteachers. Listening to the teacher, learning what he/she teaches, memorizing or constructing the answers that he/she wants to hear: Some of these skills may be useful in our professional life, but they are clearly not sufficient. When entering professional life, it is necessary to stand up, abandon that little chair, and embrace the complexity of real life. Professional objectives are usually oriented toward much more pragmatic goals: the production (as opposed to the acquisition) of knowledge, of a drug, or of a car. In recognition of this, higher‐level learning objectives should perhaps be formulated not simply as ‘the student should be able to do this’, but rather as ‘the student should be able to do this for real’. If it exists, we have not found the academic word that corresponds to this pedagogical concept. If a reader has a suggestion, please let us know. We refer to it as a ‘This is not a drill’ activity, and we have favored this type of activity throughout the curriculum. The list of ‘This is not a drill’ activities is long. It includes rather classical items (internships in research laboratories and in companies), some more playful (a microbiology course focused on fermentation and beer production), some more community‐oriented (writing a scientific data sheet for the Orphanet database on rare genetic diseases). We describe below in more detail a few examples of ‘This is not a drill’ activities which are specifically related to the scientific reading and writing objective previously described.

First‐year students start by learning the basics of scientific writing: choice of appropriate verbs, specific vocabulary, and use of short, simple, and precise sentences. In apparent contradiction with this goal, the first year (largely shared with medical students) is devoted to the acquisition of basic knowledge, a feat (sadly, but necessarily) tested by multiple‐choice questions (MCQs). The challenge was to integrate the acquisition of these writing skills into a directly useful activity for the students, preparing them for their much‐feared MCQ examinations. To achieve this goal, we train them to write MCQs on subjects taught in the first year. These MCQs are corrected on form and content by the teachers and used for practice examinations throughout the year. Students thus contribute to their own learning by jointly providing MCQs for their practice exams. Writing skills are of course also assessed in the final examination where students are asked to write MCQs on a few given topics.

Second‐year students (in groups of 5–6, assisted by two tutors) read a full scientific article, albeit a rather short and simple one, for the first time. Over a semester, they learn about the structure of an article, define its scientific background, main results and conclusions, and write a one‐page digest of the article. This digest is published in a biomedical review journal, authored by both the students and the tutors. For virtually all students, it is their first published scientific review article.

Third‐year students learn immunodetection methods. The practical part consists of testing a small number of newly produced recombinant antibodies. The results are published as technical reports coauthored by the students in a scientific journal.

Our experience is that ‘This is not a drill’ activities are perceived as meaningful and rewarding. In contrast to traditional teaching, these activities are, by nature, renewed every year. Enthusiasm does not wane either on the students’ or on the teachers’ side. The defining feature of these activities? They are fun.

One final element that separates studies from real life is the fact that in real life, one is expected to engage with real people. As we all know, real people are complicated: They are different from us, and at least from our point of view, they often appear irrational, inefficient, emotionally inadequate, and self‐centered (unlike us). Since our overarching goal is to prepare students for their future working environment, the ability to interact with colleagues is essential. We all know that a ‘good’ colleague is not just someone who is technically competent. Several social skills, including interacting harmoniously with colleagues, understanding their needs, avoiding useless conflicts or being able to manage them, and maintaining one’s inner peace in stressful situations, are essential and much sought‐after traits. This list can be turned into a series of learning objectives, and learning activities derived from these objectives. Throughout the Bachelor and Master, students learn through dedicated workshops to know themselves in order to better understand others. They identify their personal values, which gives them keys to determine their priorities and direction in life. They learn how to give constructive feedback and are introduced to nonviolent communication and to conflict management, either through role‐playing or by team‐working.

To conclude, using learning objectives to define a learning program not only modifies the content and the modalities of teaching. It also changes our role as teachers. The conventional submissive attitude of students has some advantages: For students, it provides a precise and reassuring learning environment and is less intellectually demanding; for teachers, it tends to leave less room for unexpected surprises, and to secure them in the comfortable role of an unquestioned leader. A ‘This is not a drill’ learning objective‐based program is less reassuring and more demanding for both teachers and students. As students stand up and abandon their little chairs, teachers must gradually climb down from their pedestals, stand by the students, and turn from being leaders to being companions, with only their own experience to share. Students experience an increased level of uncertainty throughout their learning activities. Teachers explore new projects, outside their specific field of knowledge. The challenges are significant, but so are the rewards: This approach is more exciting, engaging, and fulfilling, and in the end, it leaves the students better prepared for real life.

## Conflict of interest

The authors declare no conflict of interest

## Author contributions

PS and PC wrote the editorial.

